# Atrial fibrillation in vascular surgery: a systematic review and meta-analysis on prevalence, incidence and outcome implications

**DOI:** 10.2459/JCM.0000000000001533

**Published:** 2023-07-28

**Authors:** Vincenzo L. Malavasi, Federico Muto, Pietro A.C.M. Ceresoli, Matteo Menozzi, Ilaria Righelli, Luigi Gerra, Marco Vitolo, Jacopo F. Imberti, Davide A. Mei, Niccolò Bonini, Mauro Gargiulo, Giuseppe Boriani

**Affiliations:** a Cardiology Division, Department of Biomedical, Metabolic and Neural Sciences, University of Modena and Reggio Emilia, Policlinico di Modena; b Clinical and Experimental Medicine PhD Program, University of Modena and Reggio Emilia, Modena; c Vascular Surgery, Department of Medical and Surgical Sciences, University of Bologna; d Vascular Surgery Unit, IRCCS University Hospital Policlinico S. Orsola, Bologna, Italy

**Keywords:** atrial fibrillation, incidence, outcome, postoperative atrial fibrillation, prevalence, vascular surgery

## Abstract

**Aims:**

To know the prevalence of atrial fibrillation (AF), as well as the incidence of postoperative AF (POAF) in vascular surgery for arterial diseases and its outcome implications.

**Methods:**

We performed a systematic review and meta-analysis following the PRISMA statement.

**Results:**

After the selection process, we analyzed 44 records (30 for the prevalence of AF history and 14 for the incidence of POAF).

The prevalence of history of AF was 11.5% [95% confidence interval (CI) 1–13.3] with high heterogeneity (*I*^2^ = 100%). Prevalence was higher in the case of endovascular procedures. History of AF was associated with a worse outcome in terms of in-hospital death [odds ratio (OR) 3.29; 95% CI 2.66–4.06; *P* < 0.0001; *I*^2^ 94%] or stroke (OR 1.61; 95% CI 1.39–1.86; *P* < 0.0001; *I*^2^ 91%).

The pooled incidence of POAF was 3.6% (95% CI 2–6.4) with high heterogeneity (*I*^2^ = 100%). POAF risk was associated with older age (mean difference 4.67 years, 95% CI 2.38–6.96; *P* = 0.00007). The risk of POAF was lower in patients treated with endovascular procedures as compared with an open surgical procedure (OR 0.35; 95% CI 0.13–0.91; *P* = 0.03; *I*^2^ = 61%).

**Conclusions:**

In the setting of vascular surgery for arterial diseases a history of AF is found overall in 11.5% of patients, more frequently in the case of endovascular procedures, and is associated with worse outcomes in terms of short-term mortality and stroke.

The incidence of POAF is overall 3.6%, and is lower in patients treated with an endovascular procedure as compared with open surgery procedures. The need for oral anticoagulants for preventing AF-related stroke should be evaluated with randomized clinical trials.

## Introduction

Atrial fibrillation (AF) is the most common arrhythmia seen in clinical practice. In some cases, even in the absence of prior history of AF, AF may occur during an acute illness^1,2^ or may occur in a surgical setting, being more frequently documented after cardio-thoracic surgery (incidence around 30%),^3^ but also in noncardiac surgical procedures.^4^ In a recent study^5^ the rate of postoperative AF (POAF) in the cardiac surgery setting was reported to approximate 20%, whilst in noncardiac surgery it was around 1%.

The clinical assessment should also consider that history of AF and the occurrence of POAF may have long-term implications for patient outcome^6^ both in the case of cardiac surgery and in noncardiac surgery, with a potentially negative impact on survival,^3,7^ stroke and hospitalization for heart failure.^5^

Due to the higher number of noncardiac surgical interventions worldwide, AF in that setting appears to be an issue that deserves additional evaluations for the potential need for additional medical resources, increased costs and outcome implications.

As AF shares with vascular diseases several risk factors,^8^ it is not rare to meet a patient with AF with the need for a vascular interventions procedure. Nevertheless, the real weight and impact of AF in patients with arteriopathies undergoing vascular procedures or vascular surgery interventions is still unknown in terms of prevalence of a history of AF, incidence of POAF and implications for outcome. Indeed, while AF after cardiac surgery has been the object of many analyses and studies,^9–14^ and AF after general or orthopedic surgery has recently attracted some interest,^3,6,15^ very limited data are available on AF and vascular surgery.

The aim of our systematic review and meta-analysis was to evaluate the rate of history of AF and the incidence of POAF in patients undergoing vascular surgery procedures for arterial diseases and the impact of AF on outcomes, if adequately reported. When possible, endovascular surgery procedures were considered separately from open vascular surgery procedures.

## Methods

We performed a systematic review and meta-analysis following the PRISMA (Preferred Reporting Items for Systematic Reviews and Meta-Analyses) statement and checklist.^16^

A systematic literature search restricted to English full-text articles was performed through MEDLINE, Scopus, Google Scholar from 1 January 2000 to 31 March 2022.

### Search strategy, study selection, data extraction, and quality assessment

We used the following MeSH terms: ‘atrial fibrillation’ AND ‘vascular surgery’ to identify all the studies in which AF either was an anamnestic factor or was raised after surgery. No restriction was applied to study type. The whole list of MeSH terms is shown in Supplementary Materials (Table 1, Supplemental Digital Content).

Three independent researchers (F.M., P.C., M.M.) selected studies with the following criteria: history of AF or onset of a new AF; a cohort of at least 100 patients. Two senior reviewers (G.B. and V.L.M.) independently checked the study selection and the data extraction process. Discrepancies were resolved by consensus.

POAF was defined as a new-onset AF in patients without history of previous AF.^3^

We collected separately the data in two databases: studies in which AF was an anamnestic feature for the patients treated with vascular surgery procedures; studies showing postoperative AF (POAF).

The following data were retrieved: number of patients, mean age, number of females, risk factors, history of heart disease, type of surgery (i.e. open surgery or endovascular surgery procedure), time of onset of POAF, death or stroke in the follow-up. If detailed, the site of surgery, i.e. carotid, abdominal aorta or infra-inguinal arteries, was evaluated in subsequent analysis.

The quality of the data for each study was assessed using the Newcastle-Ottawa scale for nonrandomized cohort studies.^17^ We evaluated the following domains: study group selection, study group comparability, and outcome assessment; a score of ≥7 identified high-quality studies.

### End points

End points of the study were to describe: the prevalence of history of AF; the incidence of POAF; the outcome of the patients in terms of in-hospital death and stroke.

### Statistical analysis

In the two collected databases we conducted, wherever possible, analysis with two distinct techniques.

With the aim to describe the pooled prevalence/incidence of AF/POAF we conducted a meta-analysis of proportions. Prevalence/incidence was transformed using logit transformation and were pooled with the inverse variance method; tau was estimated with the restricted maximum-likelihood (REML) method.

To evaluate the impact of the history of AF on hospital mortality and onset of stroke we also performed a pairwise meta-analysis.

Moreover, where available, baseline variables were further meta-analyzed comparing AF vs. no AF patients, and were summarized as mean difference or odds ratio (ORs) and respective 95% confidence intervals (95% CIs).

All meta-analyses were modeled with a random-effect approach and results were graphically reported with forest plots. The inconsistency index (*I*^2^) was employed to measure heterogeneity among the studies for each analysis. The following thresholds were applied: low heterogeneity if *I*^2^ < 25%, moderate if *I*^2^ between 25% and 75% and high if *I*^2^ > 75%. If *I*^2^ was >25% we performed a sensitivity analysis using the ‘leave-one-out’ technique.

To account for potential sources of heterogeneity in the pooled prevalence of AF, we performed several subgroup analyses, according to the year of publication (2016 or prior vs. 2017 or after), the mean age, gender, sample size (fewer than vs. more than 5000 cases in the prevalence study and fewer than vs. more than 500 cases in the incidence one), study design (prospective vs. retrospective), site (aortic vs. carotid vs. lower limb) and technique of surgery (open vs. endovascular procedures), main comorbidities, and quality of the records (good vs. doubtful). Where specified, pooled estimates were reported as ORs and 95% CI or mean difference and 95% CI for continuous variables.

Trying to explain high heterogeneity, meta-regression analyses according to study design, sample size, mean age of the patients, risk factors, coronary artery disease, heart failure and type of surgery were performed when the number of records (*k*) carrying the information was ≥5.

Publication bias was assessed by visual inspection of funnel plots and using the Egger's test.

Statistical analysis was conducted with R v.4.1.2^18^ and its interface RStudio^19^ using meta^20^ and metafor^21^ packages.

## Results

**Fig. 1:**
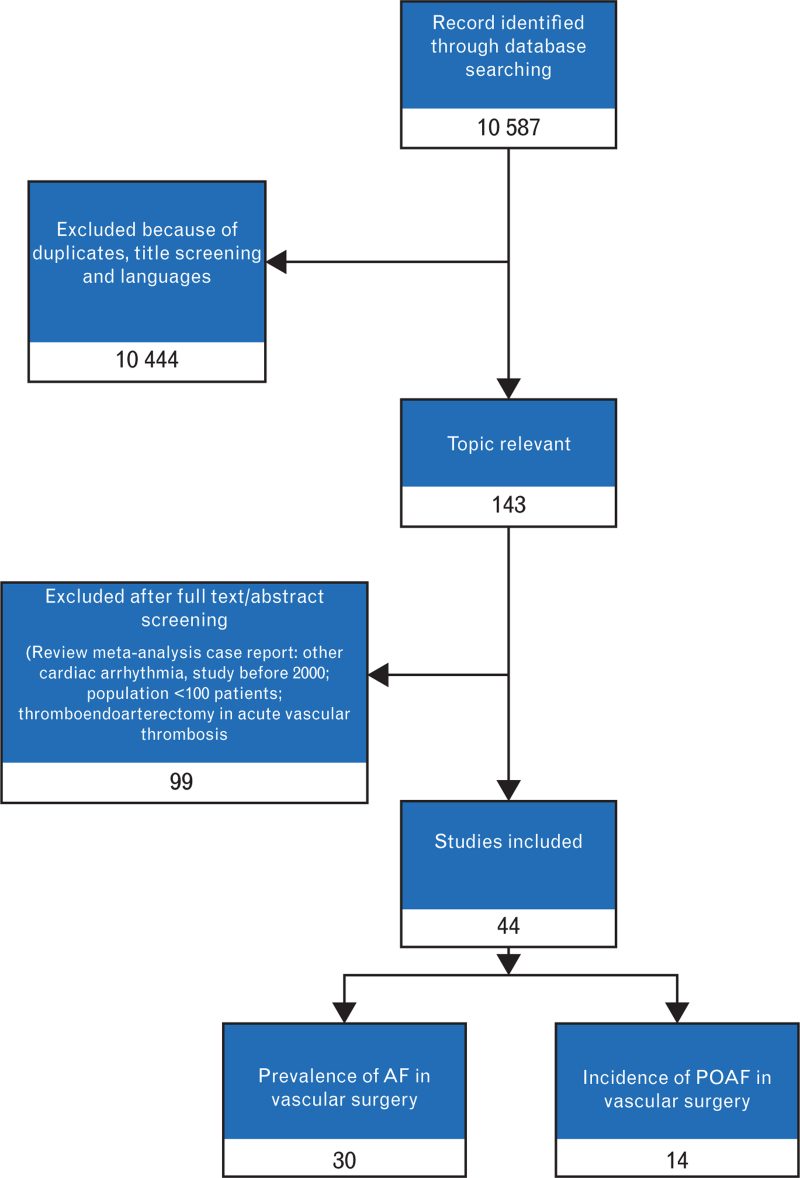
Selection process of the reports in the literature. AF, atrial fibrillation; POAF, postoperative atrial fibrillation.

From the initial 10 587 papers, after the selection process, we analyzed 44 papers. Thirty papers contribute to the first analysis on the prevalence of history of AF in patients undergoing vascular surgery and 14 were considered for the incidence of POAF. The two cohorts were representative of 3 501 739 patients for the analysis of AF prevalence and 400 771 patients in the analysis of incident AF, respectively. The selection process is shown in Fig. 1. Risk-of-bias inspection revealed that only 9 up to 36 studies were of good quality (Table 2, Supplemental Digital Content in the Supplementary Materials section).

### Prevalence of atrial fibrillation

The studies regarding history of AF were 22 retrospective^22–43^ and 8 prospective.^44–51^

A synthesis of the studies included in the analysis is shown in Table 1.

**Table 1:** Studies included in the meta-analysis evaluating the prevalence of history of AF

Author year	Study type	Total patients	Mean age	Female (%)	AF patients (%)	Vascular surgery site
Jack Tu 2003	Retrospective	6038	68.3	34.7	5.1	Carotid intervention
Harthun 2010	Retrospective	20 022	71	43.0	7.2	Carotid intervention
Van Diepen 2011	Prospective	636	NR	NR	13.2	NR
Hawkins 2012	Prospective	11 122	70.6	38.5	12.4	Carotid intervention
Sanders 2012	Retrospective	14 524	NR	14.1	11.9	Abdominal aortic intervention
Chang 2014	Prospective	253	70.9	51.0	17.8	Peripheral intervenction
Ogata 2014	Prospective	302	70.2	15.6	6.3	Carotid intervenction
Querishi 2014	Retrospective	22 177	75	48.3	10.4	Carotid intervention
Mao 2014	Retrospective	7568	71	38.0	11.3	Peripheral intervention
Sevilla 2015	Prospective	176	75.4	2.8	18.2	Abdominal aortic intervention
Saddiq 2015	Prospective	225 191	71	42.0	8.4	Carotid intervention
Watabe 2015	Retrospective	672 074	71	42.0	8.8	Carotid intervention
Huang 2016	Prospective	511	71	44.6	7.2	Peripheral intervention
Ralevic 2016	Prospective	282	68	24.5	16.3	Peripheral intervention
Behrendt 2017	Prospective	2798	70.4	38.6	16.0	Peripheral intervention
Atti 2018	Retrospective	138 014	NR	19.4	13.4	Abdominal aortic intervention
Higashitani 2018	Prospective	2238	73.3	28.5	11.0	Peripheral intervention
Huang 2019	Prospective	936	71	44.0	13.8	Peripheral intervention
Mazzaccaro 2019	Prospective	473	85	37.0	11.4	Carotid intervention
Pacha 2019	Retrospective	2 283 568	68.2	42.8	12.9	Peripheral intervention
Reis V. 2019	Prospective	928	69	20.0	6.3	Abdominal aortic, carotid and peripheral intervention
Reis P. 2020	Prospective	306	66	29.7	6.2	Abdominal aortic, carotid and peripheral intervention
Nejim 2020	Retrospective	86 778	71	42.0	7.8	Carotid intervention
D’Cruz 2020	Prospective	211	67.8	NR	19.9	Peripheral intervention
Gonzalez 2020	Retrospective	403	70.1	22.8	18.9	Peripheral intervention
Tomoi 2021	Prospective	2190	73	28.7	14.2	Peripheral intervention
Peric 2021	Prospective	144	69.9	NR	4.9	Abdominal aortic, carotid and peripheral intervention
Katsuki 2021	Prospective	911	72.9	29.0	13.5	Peripheral intervention
Honda 2021	Prospective	363	73.5	33.1	16.8	Peripheral intervention
Barenbrock 2022	Prospective	602	70.1	26.9	25.7	Peripheral intervention

AF, atrial fibrillation; NR, not reported.

**Fig. 2:**
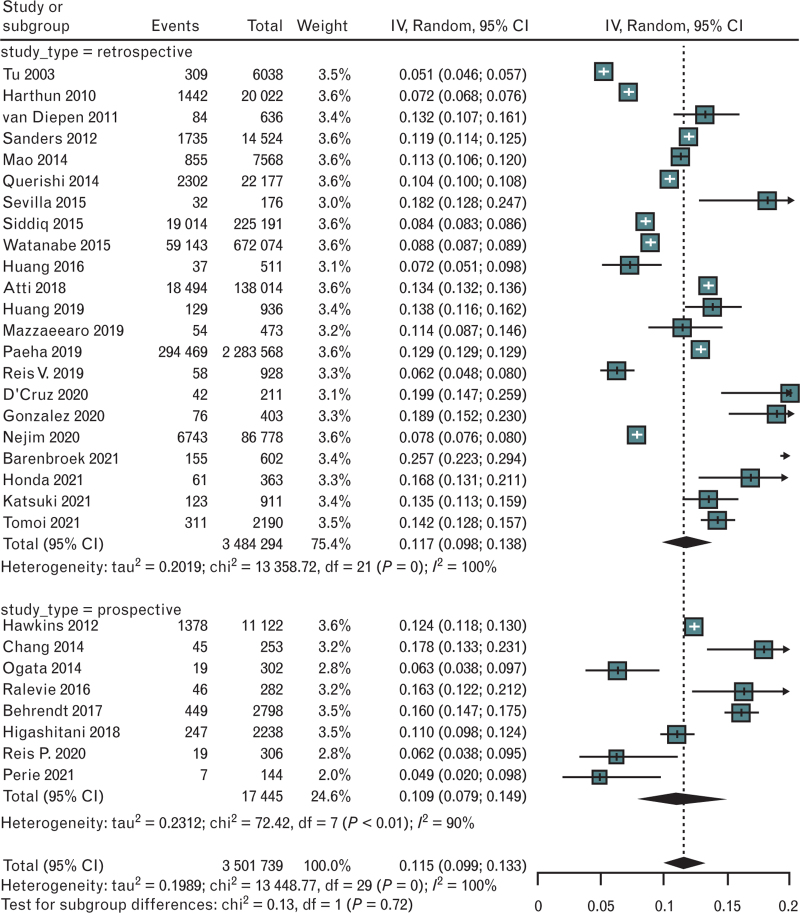
Forest plot evaluating the pooled proportion of prevalence of AF in studies about vascular surgery, according to study type. AF, atrial fibrillation.

The overall weighted proportion of history of AF was 11.5% (95% CI 1–13.3) without significant difference between retrospective [11.7% (95% CI 9.8–13.8)] and prospective studies [10.9% (95% CI 7.9–14.9)] (*P* = 0.72) (Fig. 2). We found a very high heterogeneity (*I*^2^ 100%); sensitivity analysis did not modify these findings (Figure 1 in the Supplementary Materials section).

The analysis of funnel plot and the Egger's test did not found any publication bias (Egger's test *P* = 0.314) (Figure 2, Supplemental Digital Content in the Supplementary Materials section).

To identify the source of this very high heterogeneity, we made a univariate meta-regression with mean age, gender, sample size, type of study (retrospective vs. prospective), and site of surgery as covariates. The latter only partly explained the heterogeneity (Table 3, Supplemental Digital Content in the Supplementary Materials section), where surgery or procedures on the carotid district (*P* = 0.007) and studies that included mixed interventions (aortic, lower limbs, carotid) (*P* = 0.001) significantly modified the heterogeneity when compared with aortic surgery procedures. The multivariable model, with all above covariates, added little to that univariate meta-regression (Table 3, Supplemental Digital Content in the Supplementary Materials section). However, the residual *I*^2^ is always above 90%.

With the same goal, we also made several subgroups analyses. Heterogeneity remains very high when we split the analysis for type of study (retrospective *I*^2^ = 100% vs. prospective *I*^2^ = 90%), quality of the studies (good *I*^2^ = 96% vs. doubtful *I*^2^ = 98%), sample size (>5000 cases, *I*^2^ = 100% vs. ≤5000 cases 92%), year of publication (2016 or before *I*^2^ = 98% vs. 2017 or after *I*^2^ = 99%), type of surgery (endovascular *I*^2^ = 99% vs. open surgery *I*^2^ = 100%), and site of surgery (aortic *I*^2^ = 93% vs. carotid *I*^2^ = 99% vs. lower limb *I*^2^ = 93%).

**Fig. 3:**
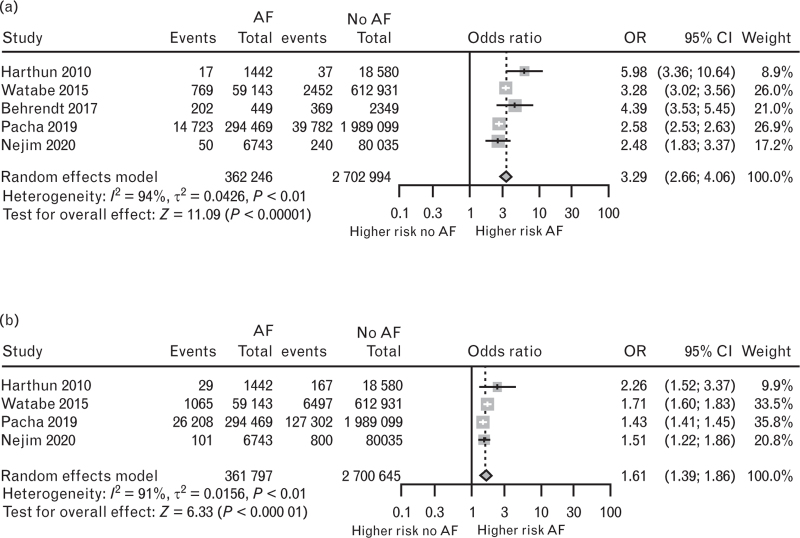
In panel (a), forest plot evaluating the association between history of AF and death; in panel (b) forest plot evaluating the association between history of AF and stroke. AF, atrial fibrillation; 95% CI, 95% confidence interval; OR, odds ratio.

**Fig. 4:**
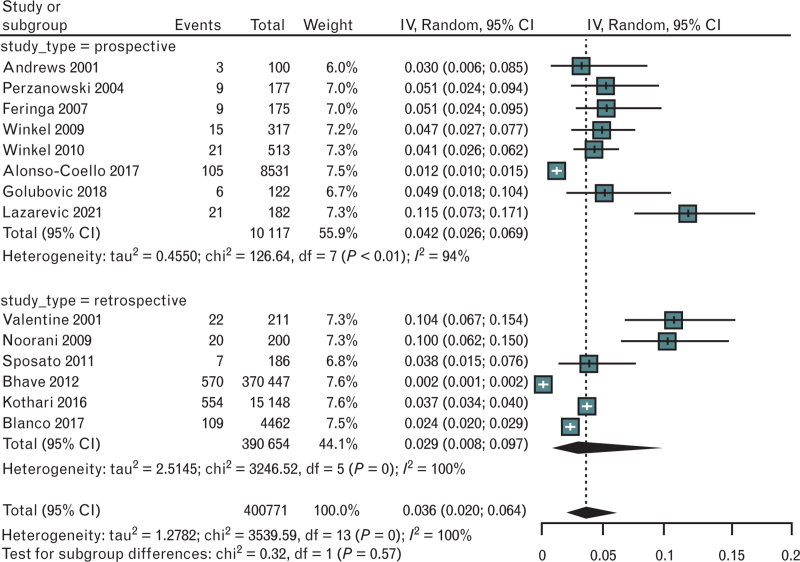
Forest plot evaluating the pooled proportion of incidence of POAF in studies about vascular surgery, according to study type. POAF, postoperative atrial fibrillation.

The weighted proportion of patients with previous AF was significantly higher in patients undergoing aortic and lower limbs surgery when compared with carotid or miscellaneous studies (aortic 13%, carotid 8%, lower limbs 15%, mixed 6%; subgroups differences *P* < 0.01) (Figure 3, Supplemental Digital Content in the Supplementary Materials section). Moreover, the pooled prevalence of history of AF was higher in patients undergoing endovascular surgery procedures when compared with those who were treated with open vascular surgery interventions (14% vs. 9.2%; *I*^2^ 100%; *P* for subgroup differences < 0.01) (Figure 4, Supplemental Digital Content in the Supplementary Materials section).

Information about hospital death and stroke was available for five and four studies, respectively. In a pooled population of 3 065 240 patients, history of AF was associated with in-hospital death (OR 3.29; 95% CI 2.66–4.06; *P* < 0.0001; *I*^2^ 94%) and in a sample of 3 062 442 patients history of AF was associated with stroke at follow-up (OR 1.61; 95%CI 1.39–1.86; *P* < 0.0001: *I*^2^ 91%) (Fig. 3a and b). It was not possible to assess the outcome of patients with history of AF in relation to the different sites of intervention for vascular surgery procedures, since only two studies in carotid surgery and one study in lower limb surgery reported these outcome data.

Considering the sources of heterogeneity, at sensitivity analysis we found that omitting the study of Pacha,^35^ evaluating in-hospital death the heterogeneity was lower (*I*^2^ = 78%) and it decreased much more (*I*^2^ = 39%) in the evaluation of stroke.

Three studies^30,35,39^ detailed the outcome in patients treated with endovascular surgery procedures separately from open vascular surgery. In both settings, history of AF was associated with a worse outcome when compared with no history of AF (in detail, the analysis on death found in endovascular surgery procedures an higher risk in AF vs. no AF: OR 2.44; 95% CI 2.37–2.51; *P* < 0.0001 and an increased risk was also found for open surgery procedures in AF vs. no AF: OR 2.77; 95% CI 2.41–3.18 *P* < 0.0001 (Figure 5, Supplemental Digital Content in the Supplementary Materials section). Also, the analysis on stroke highlighted an increased risk in patients with AF, both in endovascular surgery procedures (OR 1.34; 95% CI 1.32–1.37; *P* < 0.0001) and in open surgery interventions (OR 1.59; 95% CI 1.37–1.85; *P* < 0.0001) (Figure 6, Supplemental Digital Content in the Supplementary Materials section).

### Incidence of postoperative atrial fibrillation

Information about incident AF after vascular surgery procedures was found in 14 studies: 8 prospective^52–59^ and 6 retrospective.^60–65^

No study reported specific data about the outcome in incident AF patients, and therefore we analyzed only the rates of reported POAF across the studies. A synthesis of included studies is shown in Table 2.

**Table 2:** Studies included in the meta-analysis evaluating the incidence of POAF

Author year	Study type	Total patients	Vascular surgery (%)	Mean age	Site vascular surgery	POAF (%)
Andrews 2001	Prospective	100	100.0	70.5	Abdominal aortic, peripheral and carotid intervention	3.00
Valentine 2001	Prospective	211	100.0	66	Abdominal aortic intervention	10.43
Perzanowski 2004	Prospective	177	50.8	67.6	Abdominal aortic intervention	10.00
Feringa 2007	Prospective	175	100.0	NR	Abdominal aortic intervention	5.14
Noorani 2009	Prospective	200	100.0	70	Abdominal aortic intervention	10.00
Winkel 2009	Prospective	317	100.0	68.6	Abdominal aortic and peripheral intervention	4.73
Winkel 2010	Prospective	513	100.0	68.6	Abdominal aortic, peripheral and carotid intervention	4.09
Sposato 2011	Prospective	186	100.0	68.6	Carotid intervention	3.76
Bhave 2012	Retrospective	370 447	5.7	62.7	Abdominal aortic, peripheral and carotid intervention	NR
Kothari 2016	Retrospective	15 148	100.0	73.7	Abdominal aortic intervention	3.66
Blanco 2017	Retrospective	4462	100.0	63.8	Abdominal aortic intervention	2.44
Alonso-Coello 2017	Prospective	8531	40.6	70	Abdominal aortic, peripheral and carotid intervention	3.03
Golubovic 2018	Prospective	122	100.0	67	Abdominal aortic, peripheral and carotid intervention	4.92
Lazarevic 2021	Prospective	182	100.0	67.2	Abdominal aortic, peripheral and carotid intervention	11.54

NR, not reported; POAF, postoperative atrial fibrillation.

The pooled incidence of POAF was 3.6% (95% CI 2–6.4) without differences between retrospective vs. prospective studies (*P* = 0.57) (Fig. 4). The degree of heterogeneity was very high (*I*^2^ 100%), but at the leave-one-out analysis we did not identify a study able to significantly modify the heterogeneity (Figure 7, Supplemental Digital Content in in the Supplementary Materials section). The funnel plot and the Egger's test found a significant risk-of-bias (Egger's test *P* = 0.03; Figure 8, Supplemental Digital Content in the Supplementary Materials section).

POAF risk was associated with older age (weighted mean difference 4.67 years, 95% CI 2.38–6.96; *P* = 0.00007 – standardized mean difference 0.47, 95% CI 0.26–0.69; *P* < 0.0001) (Table 3).

**Table 3:** Results of analysis of the covariates associated with POAF. Data were expressed as odds ratios and 95% confidence intervals except for age where the weighted mean difference in years was accounted for

	OR	95% CI	*P*	*I* ^2^	*k*
Age (MD)	4.67	2.38–6.96	<0.0001	84%	8
Female sex	1.16	0.72–1.76	0.45	48%	7
Hypertension	1.06	0.90–1.25	0.49	0%	8
Diabetes	1.05	0.77–1.44	0.74	22%	7
CAD	1.18	0.60–2.08	0.72	56%	7
HF	1.89	0.38–9.32	0.43	79%	4
Stroke	1.66	0.58–4.75	0.34	64%	4

CAD, coronary artery disease; CI, confidence interval; HF, heart failure; *I*^2^, heterogeneity according to *I*^2^ index; *k*, number of studies; MD, mean difference; OR, odds ratio; POAF, postoperative atrial fibrillation.

Female sex (OR 1.16; 95% CI 0.72–1.76; *P* = 0.45), history of hypertension (OR 1.06; 95% CI 0.90–1.25; *P* = 0.49), diabetes (OR 1.05; 95% CI 0.77–1.44; *P* = 0.74), coronary artery disease (CAD) (OR 1.12; 95% CI 0.60–2.08; *P* = 0.72), HF (OR 1.89; 95% CI 0.38–9.32; *P* = 0.43) and stroke (OR 1.66; 95% CI 0.58–4.75) were not significantly associated with POAF (Table 3).

At a univariable meta-regression, sample size, female sex, and history of CAD significantly modified heterogeneity, but only history of CAD and a multivariable meta-regression, with the three covariates mentioned above, significantly modified the residual heterogeneity (residual *I*^2^ respectively 36% and 51%) that, after the regression, resulted as no longer statistically significant (Table 4, Supplemental Digital Content in the Supplementary Materials section).

Considering the differences between open surgery and endovascular surgical procedures, whenever not indicated what specific approach was used, we considered every intervention to be an open vascular surgery procedure. The pooled incidence of POAF was higher in the patients treated with an open surgical procedure as compared with an endovascular surgical procedure (8.2% vs. 2.4%; p for subgroups difference < 0.01) (Figure 9, Supplemental Digital Content in the Supplementary Materials section). In three studies, the information on the type of approach (endovascular or open vascular surgery) was very detailed and allowed a pairwise comparison; this analysis confirmed that POAF was less likely to be associated with endovascular surgical procedures as compared with open surgery (OR 0.35; 95% CI 0.13–0.91; *P* = 0.03) (Figure 10, Supplemental Digital Content in the Supplementary Materials section).

With regard to potential differences in POAF incidence in different sites of surgery, the analysis had important limitations since few studies were available with detailed information on carotid (two studies ^59,62^) and lower limb (one study^59^) surgery. Given this necessary premise, an explorative analysis done with available data, showed no difference in POAF according to the site of vascular surgery interventions (Figure 11, Supplemental Digital Content in the Supplementary Materials section).

## Discussion

The significance of AF in patients undergoing vascular surgery interventions or endovascular procedures for arterial diseases has not been the object of a comprehensive assessment.

Our study extends the knowledge in the field by highlighting that: the prevalence of history of AF in patients who are candidates for vascular surgery is 11.5%%; patients with a history of AF treated with a vascular surgery intervention have a worse outcome in terms of stroke and death as compared with patients with no history of AF; the incidence of POAF in patients who have had a vascular surgery intervention is 3.6%; the prevalence of history of AF is higher in patients undergoing endovascular surgery procedures when compared with those who are treated with open vascular surgery interventions, while the pooled incidence of POAF is higher in patients treated with an open surgical procedure.

Data about this topic are definitely lacking and our work almost always consisted of a hunt looking for vascular patients in broader general surgery cohorts. This limitation leads to limitations in data quality (as documented in Table 2, Supplemental Digital Content in the Supplementary Materials section), but at the same time covers an area of uncertainty in the literature.

### Prevalence of history of atrial fibrillation in vascular surgery and related outcomes

AF is a major issue in cardiac and noncardiac surgery^8^ but this issue is poorly analyzed and reported in the specific setting of vascular surgery.

In noncardiac surgery, prevalence of AF is about 4–7%,^66,67^ and comparable in a very specific subset of patients, such as patients undergoing orthotopic liver transplant.^68^

A previous meta-analysis, published in 2017, evaluating patients with symptomatic PAD^69^ and including prospective cohort studies took into account patients categorized according to presence or absence of AF (electrocardiographic evidence of arrhythmia) at the time of enrollment. In that study, which was not focused on surgical interventions, but simply on PAD, the average prevalence of AF among PAD patients was 11.4% (range, 8.0%–17.9%). Our study shows that in the setting of vascular surgery, the prevalence of history of AF is 11.5% (95%CI 9.9–13.3), although with very high heterogeneity among the studies. The only explanation we found for substantial heterogeneity was the type of surgery. Indeed the weighted proportion of patients with history of AF was 6% in studies analyzing different types of vascular surgery, 8% in carotid procedures, 13% in aortic surgery and 15% in lower limb surgery (Figure 4, Supplemental Digital Content in the Supplementary Materials section). It is noteworthy to consider that the approach to the vascular pathology may be endovascular or a traditional open vascular surgery.

According to literature among patients undergoing noncardiac surgery, those with a history of AF were at higher risk of cardiovascular events,^66^ as well as a higher risk of mortality and stroke at 30 days.^70^

However, for a detailed interpretation of all these data, an important role could be played by the preoperative cardiological evaluation and antithrombotic therapy, in both the pre and postoperative periods, but these data are usually lacking.

In our analysis, we found that preoperative history of AF was associated with a higher likelihood of in-hospital death and stroke, although with high heterogeneity suggesting the need for further studies trying to better target the patients at higher risk, also from the perspective of preventive treatments. The rate of these events appears higher as compared with the patients with PAD.^71^ A variable approach to AF management in the pre and postoperative periods could explain the high heterogeneity of our findings. Additionally, the association of AF with a series of comorbidities^72,73^ has to be considered when evaluating the worse outcome associated with AF.

A meta-analysis of studies comparing open vascular surgery with endovascular treatment for femoropopliteal arterial disease showed that femoral bypass surgery was associated with higher morbidity (OR 2.93; 95% CI 1.34–6.41) but similar mortality at 30 days compared with endovascular treatment.^74^ However, in that study AF prevalence and antithrombotic therapy management were not reported.

We found that the prevalence of history of AF was higher in patients treated with endovascular procedures; this finding is more likely related to the selection of patients. Only a few studies reported detailed data on the outcome of patients with vs. without history of AF in the different vascular surgery settings. We found that, in general, patients with a history of AF had a significantly worse outcome with a more than double probability of death and also a significantly increased risk of developing a stroke. This was also confirmed when studies on endovascular surgery and studies on open surgery were analyzed. According to these findings it is clear that AF may be a marker of increased risk that can be explained by the higher clinical complexity typical of AF patients.^75,76^

### Incident atrial fibrillation after vascular surgery

According to our data vascular surgery for arterial diseases is the noncardiac surgery setting where POAF shows a quite high incidence^77^ similar to thoracic surgery. Interestingly, in a recent meta-analysis^4^ that excluded patient treated with vascular surgery interventions, the incidence of POAF was 1.7% while we found an almost double pooled incidence of POAF (3.6%; 95% CI 2.0–6.4).

We found that the incidence of POAF was higher in patients treated with open surgery vs. endovascular approaches. To interpret this finding, it is probable that a series of peri-operative factors and complications, as well as a process of inflammation related to surgery, may act as a trigger for AF.^78–80^

In patients treated with vascular surgery procedures for arterial diseases, the most significant risk factor associated with POAF was weighted mean difference in age, with POAF patients 4.6 years older than patients without POAF. This finding is in line with the epidemiology of AF, characterized by increasing risk of AF at increasing age.^72,81^ However, it should be noted that the available data (Table 3) were limited and there was a notable absence of structured analysis on cardiovascular risk factors, with only a minority of studies providing such information.

Our meta-analysis did not allow the outcome of patients presenting POAF after a vascular surgery procedure to be assessed, but a higher incidence of stroke/systemic embolism could be expected, in line with what was found in unselected patients undergoing noncardiac surgery, according to a retrospective study from Korea.^82^

The risk of stroke may be related to AF recurrences, occurring during follow-up, reported in 41% of patients at 5 years, despite the initial attribution to transient risk factors (including the occurrence in a postoperative period).^83^ Indeed, the high recurrence rate suggests that POAF should not be considered as a merely transient event, a concept that dominated in the past,^2,84^ but, rather, should be analyzed as the expression of an altered atrial substrate related to an underlying atrial cardiomyopathy that sooner or later will lead to AF recurrences even without the facilitating effect or ‘transient’ risk factors such as surgery.^85–87^

Apart from the role of atrial cardiomyopathy, the profiles of patients undergoing vascular surgery affect the risk of developing AF and this can also be predicted by comorbidities (diabetes, hypertension, ischemic heart disease) which concur with assessing a ‘virtual’ CHA_2_DS_2_VASc. As is known, the higher the CHA_2_DS_2_VASc, the higher the risk of developing AF in the long term in various subsets of patients.^88–90^

There is a need to assess the impact of OAC on the risk of stroke after noncardiac surgery. In an observational study the long-term risk of thromboembolism was similar in patients with POAF and nonvalvular AF and anticoagulation therapy was associated with a comparably lower risk of thromboembolic events in patients with POAF compared with no anticoagulation therapy.^77^ In the 2020 ESC guidelines on AF^91,92^ indication for OAC is class IIa for POAF after noncardiac surgery and IIb for POAF after CABG surgery. These recommendations, however, are derived from expert opinion and do not clarify if anticoagulation should be prescribed in the long term or for a short period of time. Well conducted randomized trials are needed to answer this question.

## Limitations

In consideration of the observational nature of the studies included in this meta-analysis, our study is subject to publication bias because of the possibility that some data were not published. In our case the search was particularly difficult because we often had to extrapolate information from a number of studies evaluating a wide spectrum of noncardiac surgery interventions, with vascular surgery procedures as one of the various types of surgical interventions. Moreover, the quality of studies inserted in the paper was not always high thus reducing the strength of results.

There is a risk, in our analysis as in many similar studies in the field, that the number of POAF patients could be underestimated in relation to different and variable protocols for postoperative ECG monitoring among the included articles.

Another limitation is linked to the lack of comparative studies evaluating AF history and the incidence of POAF in specific sites of vascular surgery interventions, with detailed analysis of carotid vs abdominal aorta vs. infra-inguinal.

We also found a significant risk-of-bias in the analysis of the pooled incidence of POAF (Egger's test *P* = 0.03; Figure 5, Supplemental Digital Content in the Supplementary Materials section). This finding can be considered in our opinion of secondary importance since our exploratory question was focused on the pooled incidence of AF and not on the efficacy of AF treatments.^93^ Moreover, the power of Egger's test when there is heterogeneity can be considerably lowered. This suggests that an inspection of the funnel plot may be preferable to Egger's test when there is substantial heterogeneity.^94^

Anyway, the main limitation of the study is the high heterogeneity found in our analysis. With regard to AF prevalence, it was not possible to identify a covariate that could explain the prevalence values, neither in the sensitivity analysis, nor in the subgroups analysis, nor in meta-regression. Therefore, it was simply possible to provide a range of AF prevalences, from about 5% to 25%.

Taking into account patients’ outcomes, the high heterogeneity is explained by the intrinsic variability of the studies. Indeed, omitting the study of Pacha *et al.*,^35^ the overall heterogeneity significantly decreased, thus restoring some strength to the conclusions about patients’ outcomes.

About the incidence of POAF, the high heterogeneity can be explained by some features of the records, since the residual heterogeneity after the meta-regression is moderate when the moderator is the prevalence of CAD or a combination of sample size, female gender or history of CAD.

## Conclusions

In patients treated with vascular surgery interventions, a history of AF is not a marginal finding, since it is present in around 1 out of 10 patients, more frequently in the case of endovascular procedures, and is associated with a worse outcome in terms of short-term mortality and stroke. The incidence of postoperative newly diagnosed AF is also high, apparently higher than POAF seen in noncardiothoracic nonvascular surgery interventions and is higher in patients treated with an opensurgical procedure as compared with endovascular procedures.

According to these data, the key question is when and for how long there is a need for oral anticoagulants, but an appropriate answer should come from well planned randomized clinical trials.

## Acknowledgements

Funding. This research did not receive any specific grant from funding agencies in the public, commercial, or not-for-profit sectors.

Funding: No specific funding was available for this contribution.

Data availability statement: The data underlying this article are available in the article and in its online supplementary Material.

### Conflicts of interest

G.B.: small speaker's fees from Bayer, Boehringer Ingelheim, Boston, Daiichi-Sankyo, Janssen and Sanofi, not related with the current work. The other authors declare no conflicts of interest.

## Supplementary Material

Supplemental Digital Content
