# Supplementary material for: Atrial fibrillation in vascular surgery: a systematic review and meta-analysis on prevalence, incidence and outcome implications
Source: J Cardiovasc Med (Hagerstown). 2023 Jul 28;24(9):612–24. doi: 10.2459/JCM.0000000000001533 (PMC10754485; doi:10.2459/JCM.0000000000001533)

Atrial fibrillation in vascular surgery: a systematic review and meta-analysis on prevalence, incidence and outcome

**Vincenzo L Malavasi^1^, Federico Muto^1^, Pietro A. C. M. Ceresoli^1^, Matteo Menozzi^1^, Ilaria Righelli^1^, Luigi Gerra^1^, Marco Vitolo^1, 2^, Jacopo F. Imberti^1,2^, Davide A. Mei^1^, Niccolò Bonini^1,2^, Mauro Gargiulo^3, 4^ , and Giuseppe Boriani^1^ ^#^**

^1^Cardiology Division, Department of Biomedical, Metabolic and Neural Sciences, University of Modena and Reggio Emilia, Policlinico di Modena, Modena, Italy

^2^Clinical and Experimental Medicine PhD Program, University of Modena and Reggio Emilia, Modena, Italy

^3^ Vascular Surgery, Department of Medical and Surgical Sciences, University of Bologna, Italy

^4^Vascular Surgery Unit, IRCCS University Hospital Policlinico S. Orsola, Bologna, Italy

#

# Supplementary Materials

**Web Table 1:** List of MeSH terms used for the search:

(“atrial fibrillation”[MeSH Terms] OR (“atrial”[All Fields] AND “fibrillation”[All Fields]) OR “atrial fibrillation”[All Fields]) AND (“vascular surgical procedures”[MeSH Terms] OR (“vascular”[All Fields] AND “surgical”[All Fields] AND “procedures”[All Fields]) OR “vascular surgical procedures”[All Fields] OR (“vascular”[All Fields] AND “surgery”[All Fields]) OR “vascular surgery”[All Fields]).

**Web Table 2:** Newcastle-Ottawa scale of all the studies carrying information about AF and vascular surgery (either prevalence and incidence).

|  | **Selection** | | | | **Comparability** | **Outcome** | | |  |
| --- | --- | --- | --- | --- | --- | --- | --- | --- | --- |
| **Author**  **Year** | Representativeness of the exposed cohort | Selection of the non exposed cohort | Ascertainment of exposure | Demonstration that outcome of interest was not present at start of study | Comparability of cohorts on the basis of the design or analysis | Assessment of outcome | Was follow-up long enough for outcomes to occur | Adequacy of follow up of cohorts | **TOTAL** |
| Andrews 2001 | * | * | * | * | * | * |  |  | ****** |
| Valentine 2001 | * | * | * | * | * | * | * |  | ******* |
| Perzanowski 2004 | * | * | * | * | * | * | * |  | ******* |
| Feringa  2007 | * | * | * | * | * | * |  | * | ******* |
| Noorani  2009 | * | * |  |  | * |  |  |  | **** |
| Winkel  2009 | * | * |  | * | * | * | * |  | ****** |
| Winkel  2010 | * | * | * | * | * | * | * | * | ******** |
| Sposato  2011 | * | * | * | * | * | * | * |  | ******* |
| Bhave  2012 | * | * | * | * | * | * |  |  | ****** |
| Kothari  2016 | * | * | * | * | * | * |  |  | ****** |
| Blanco  2017 | * | * | * | * | * | * |  | * | ******* |
| Alonso-Coello 2017 | * | * | * |  | * |  | * | * | ****** |
| Golubovic 2018 | * | * | * |  | * |  |  |  | **** |
| Lazarevic 2021 | * | * | * |  | * | * | * | * | ****** |
| Jack Tu  2003 | * | * | * | * | * | * |  |  | ****** |
| Harthun  2010 | * | * | * |  | * | * |  |  | ***** |
| Van Diepen 2011 | * | * | * |  | * | * |  |  | ***** |
| Hawkins  2012 | * | * | * |  | * | * |  | * | ****** |
| Sanders  2012 |  | * |  |  | * | * |  |  | *** |
| Chang  2014 | * | * | * |  | * |  | * |  | ***** |
| Ogata  2014 |  | * | * |  | * | * | * |  | ***** |
| Querishi  2014 | * | * | * |  | * | * | * |  | ****** |
| Mao  2014 | * | * | * |  | * | * | * |  | ****** |
| Sevilla  2015 | * | * | * |  | * | * |  |  | ***** |
| Saddiq  2015 | * |  | * |  | * |  |  |  | *** |
| Watabe  2015 | * | * | * |  | * | * | * |  | ****** |
| Huang  2016 |  |  | * |  | * | * | * |  | **** |
| Ralevic  2016 | * | * | * |  | * | * | * |  | ****** |
| Behrendt 2017 | ** | * | * |  | ** | * |  |  | ******** |
| Atti  2018 | ** | * | ** |  | ** | ** |  |  | ********* |
| Higashitani 2018 | * | * | * |  | * | * | * |  | ****** |
| Huang  2019 | * | * |  |  | * | * | * |  | ***** |
| Mazzaccaro 2019 | * |  | * |  | * | * | * |  | ***** |
| Pacha  2019 | * | * | * |  | * | * | * | * | ******* |
| Reis V.  2019 | * |  |  |  | * | * |  |  | *** |
| Reis P.  2020 | * | * |  |  | * | * | * |  | ***** |
| Nejim  2020 | * | * | * |  | * | * | * |  | ****** |
| D'Cruz  2020 |  | * | * |  | * | * |  |  | **** |
| Gonzalez 2020 | * | * | * |  | * | * | * |  | ****** |
| Tomoi  2021 |  | * | * |  | * | * | * |  | ***** |
| Peric  2021 |  | * | * |  | * | * | * |  | ***** |
| Katsuki  2021 | * |  | * |  |  | * |  |  | **** |
| Honda  2021 | * | * | * |  | * | * | * |  | ****** |
| Barenbrock 2021 | * | * | * |  | * | * |  |  | ***** |

**Web Table 3:** Results of meta-regression evaluating the prevalence of AF in patients underwent a vascular procedure.


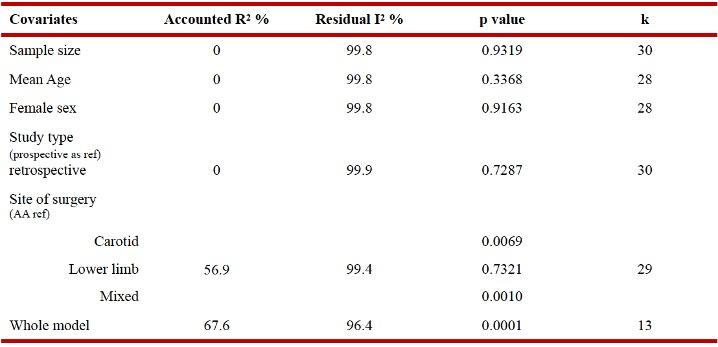


*Legend: k: number of studies; ref: reference; AA: abdominal aortic surgery. NB: mixed means various type of vascular surgery/procedures.*

**Web Table 4** Results of meta-regression evaluating the incidence of POAF in patients underwent a vascular procedure.


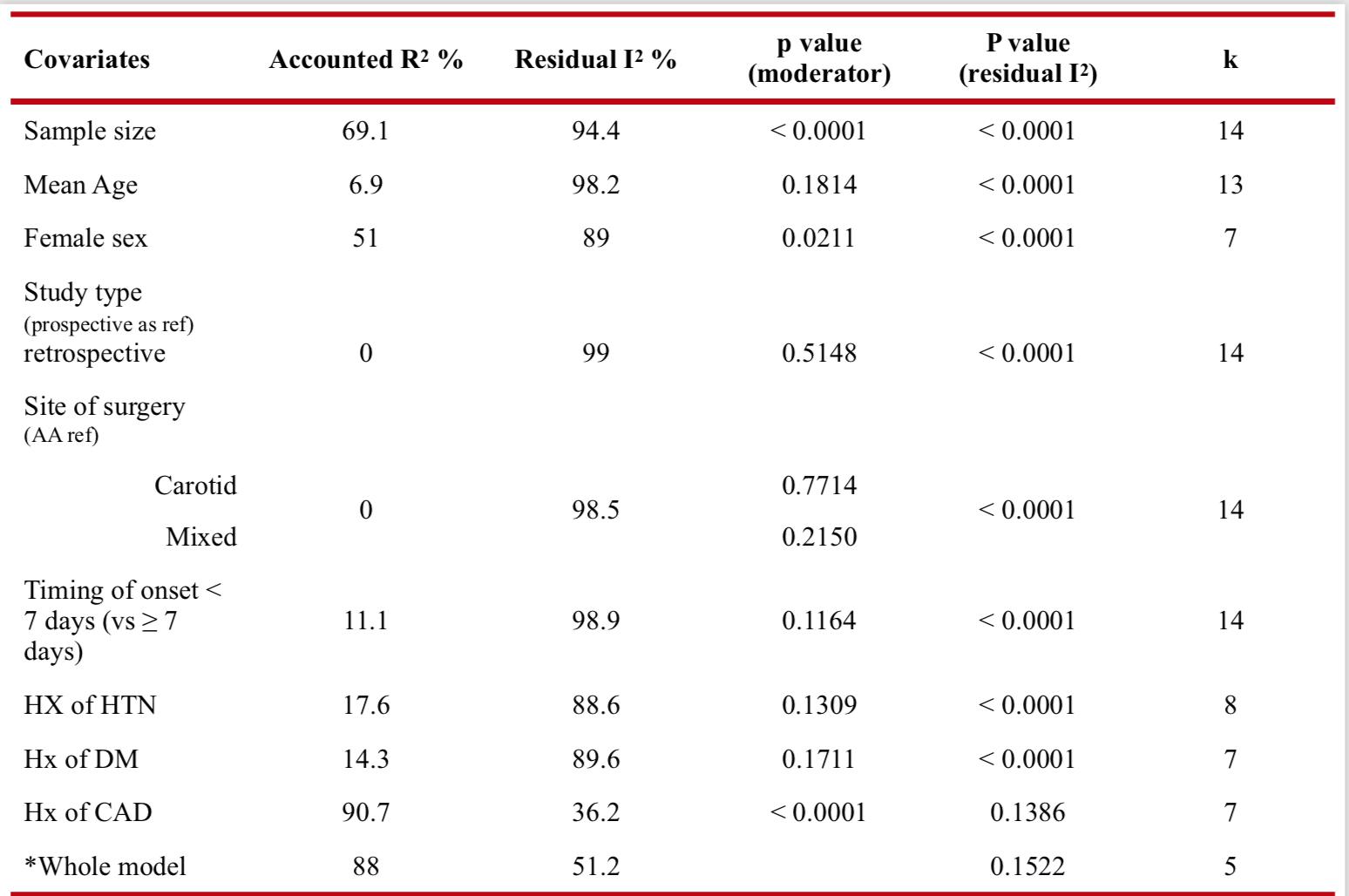


*Legend: k: number of studies; ref: reference; AA: abdominal aortic surgery. NB: mixed means various type of vascular surgery/procedures. * The whole model is a multivariable metaregression in which were inserted significant variables at uniregression analysis*

**Web Figure 1:** Leave-one-out analysis of sensitivity in studies regarding the prevalence of AF in patients treated with vascular surgery interventions.


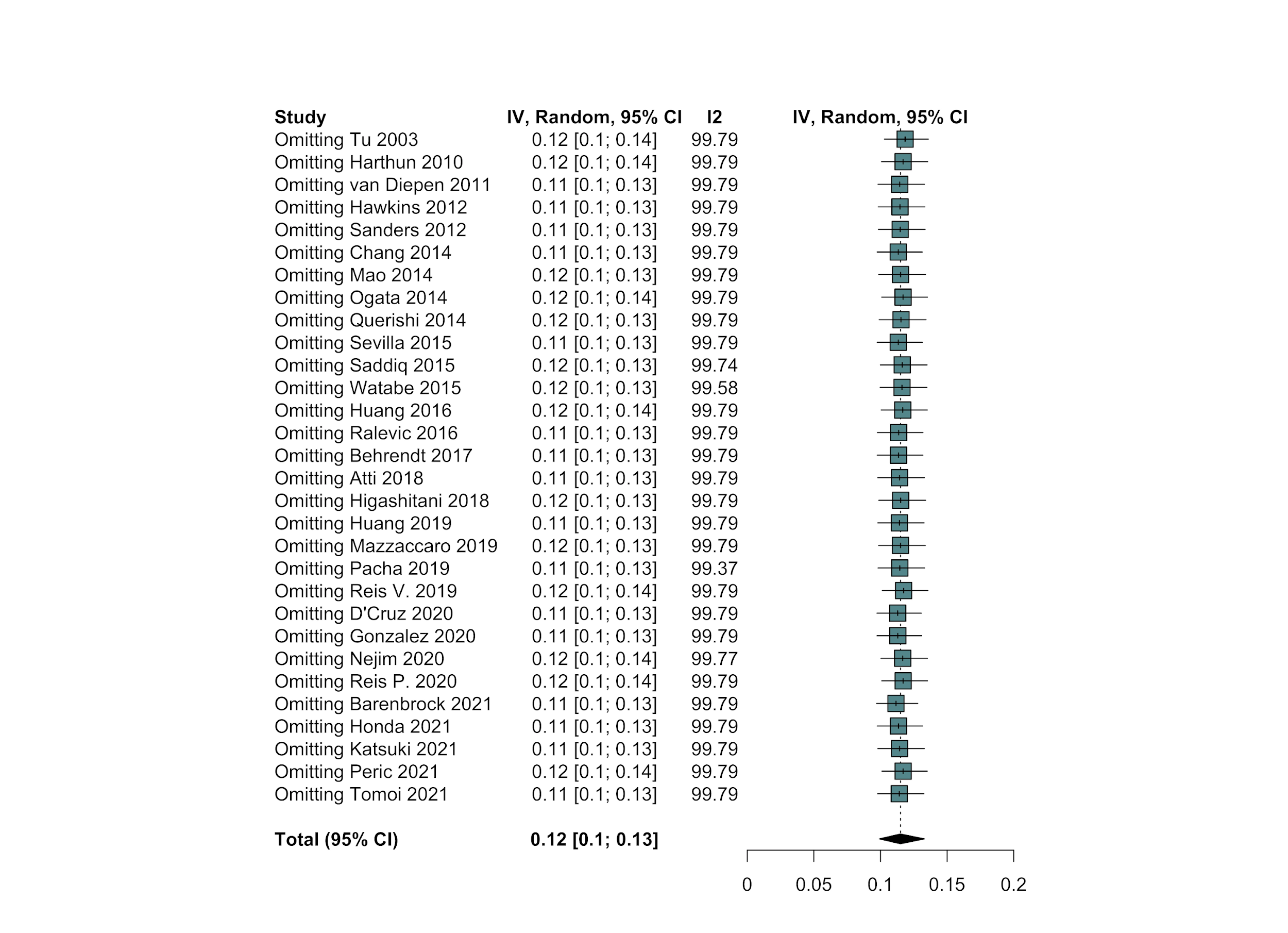


**Web Figure 2:** Funnel plot describing publication bias analyzed with Egger’s test about the prevalence of AF in vascular surgery.


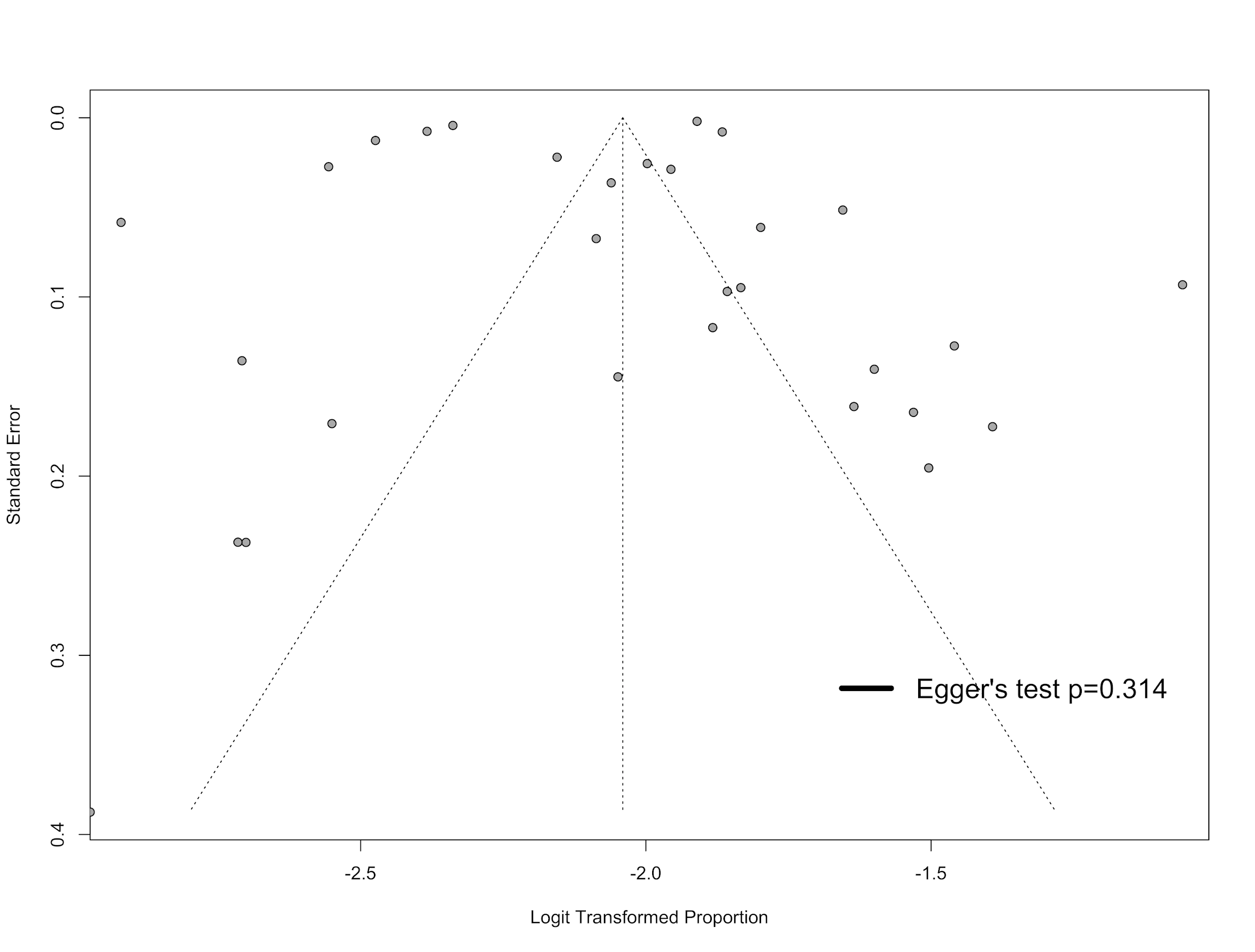


**Web Figure 3:** Analysis of prevalence of history of AF across the type of surgery. Of note the differences about carotid and mixed type of surgery where the prevalence of AF is significantly lower.


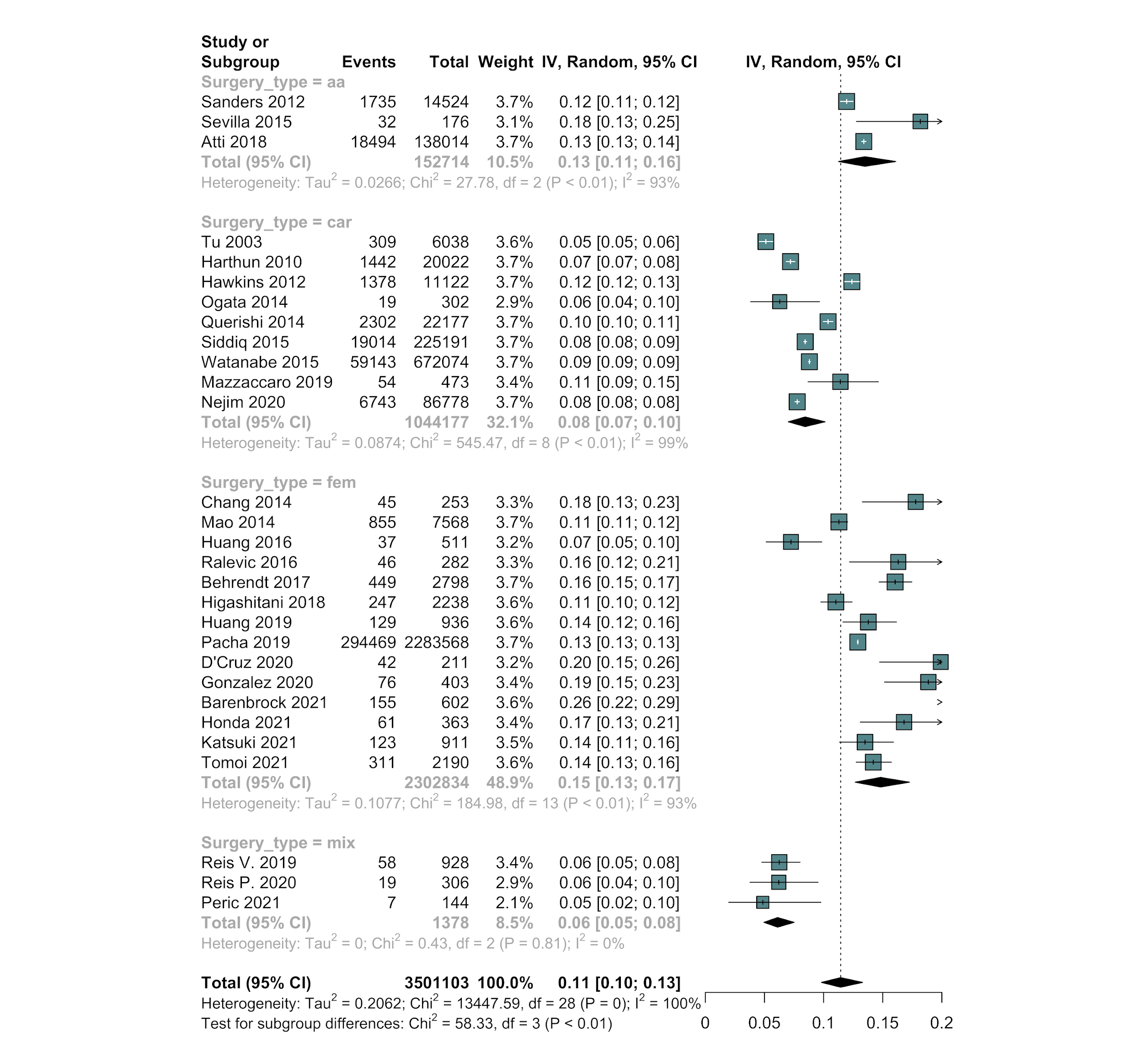


*Legend: aa = aortic surgery; car= carotid surgery; fem = lower limb vascular surgery; mix = combination of different types of vascular surgery*

**Web Figure 4:** Pooled prevalence of history of AF in endovascular surgery procedures (top) and vascular surgery interventions (bottom), respectively.


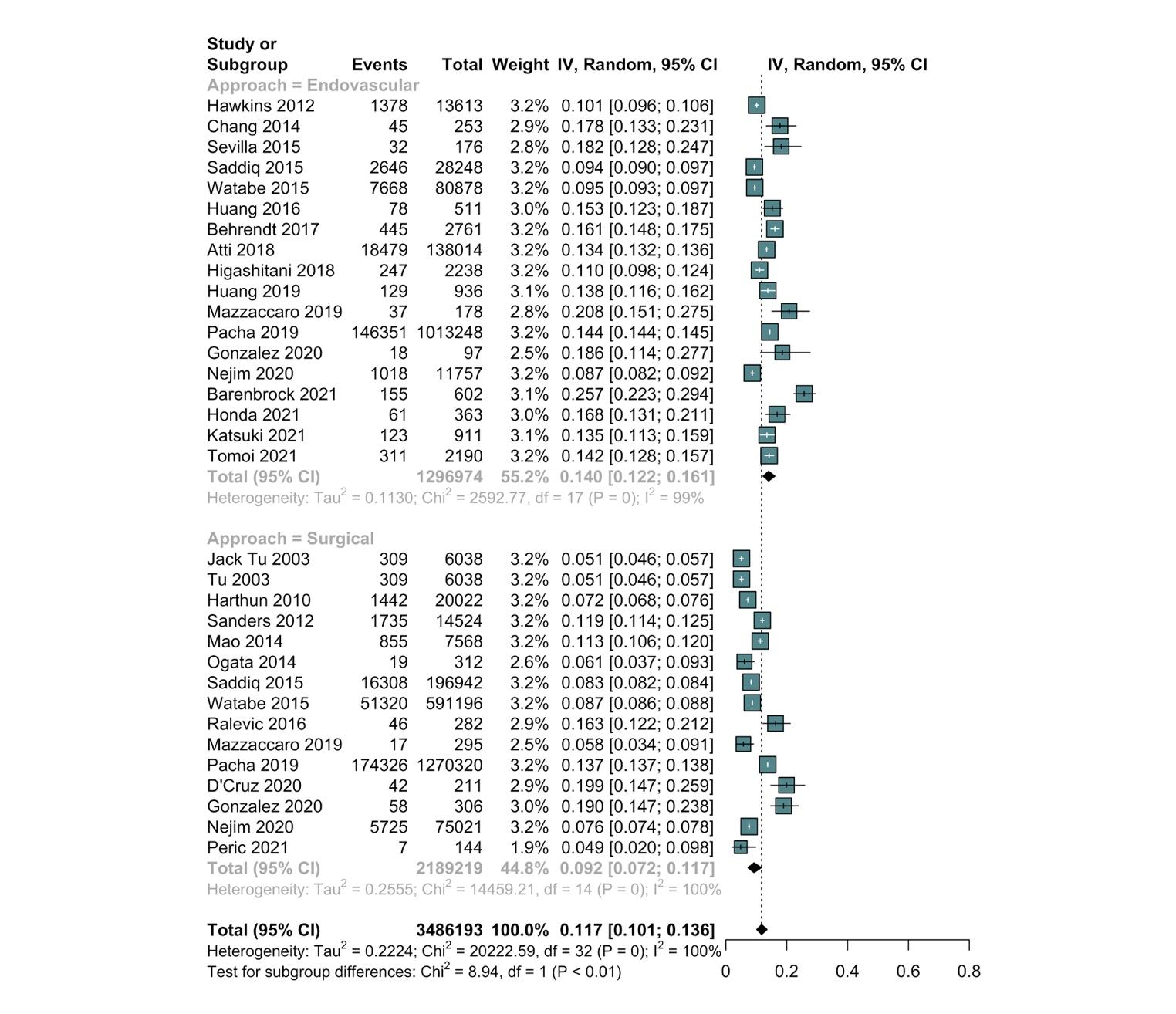


**Web Figure 5:** Risk of death in patients treated with endovascular surgery procedures (top panel) and in patients treated with open vascular surgery (bottom panel) according to AF presence.


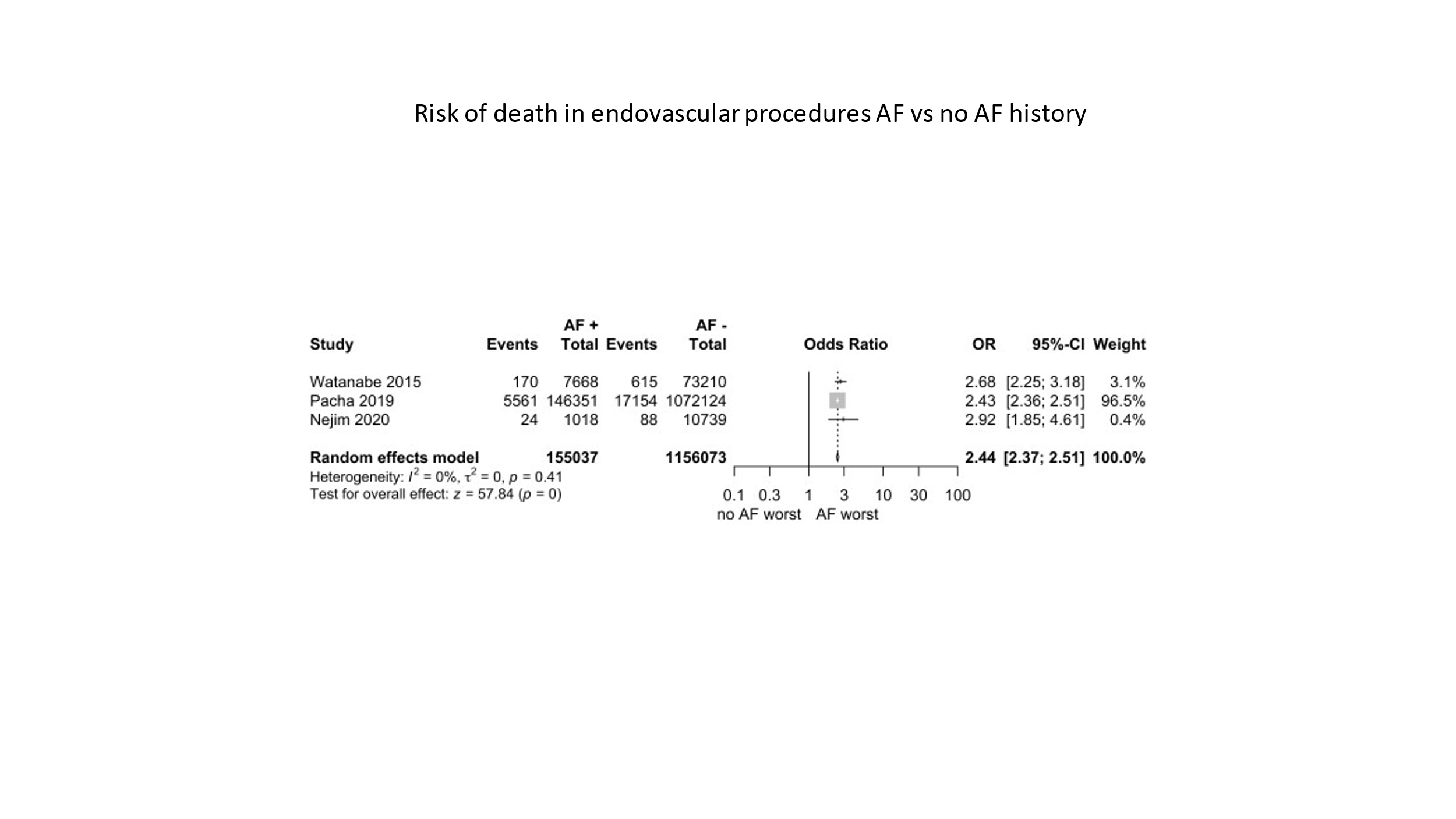


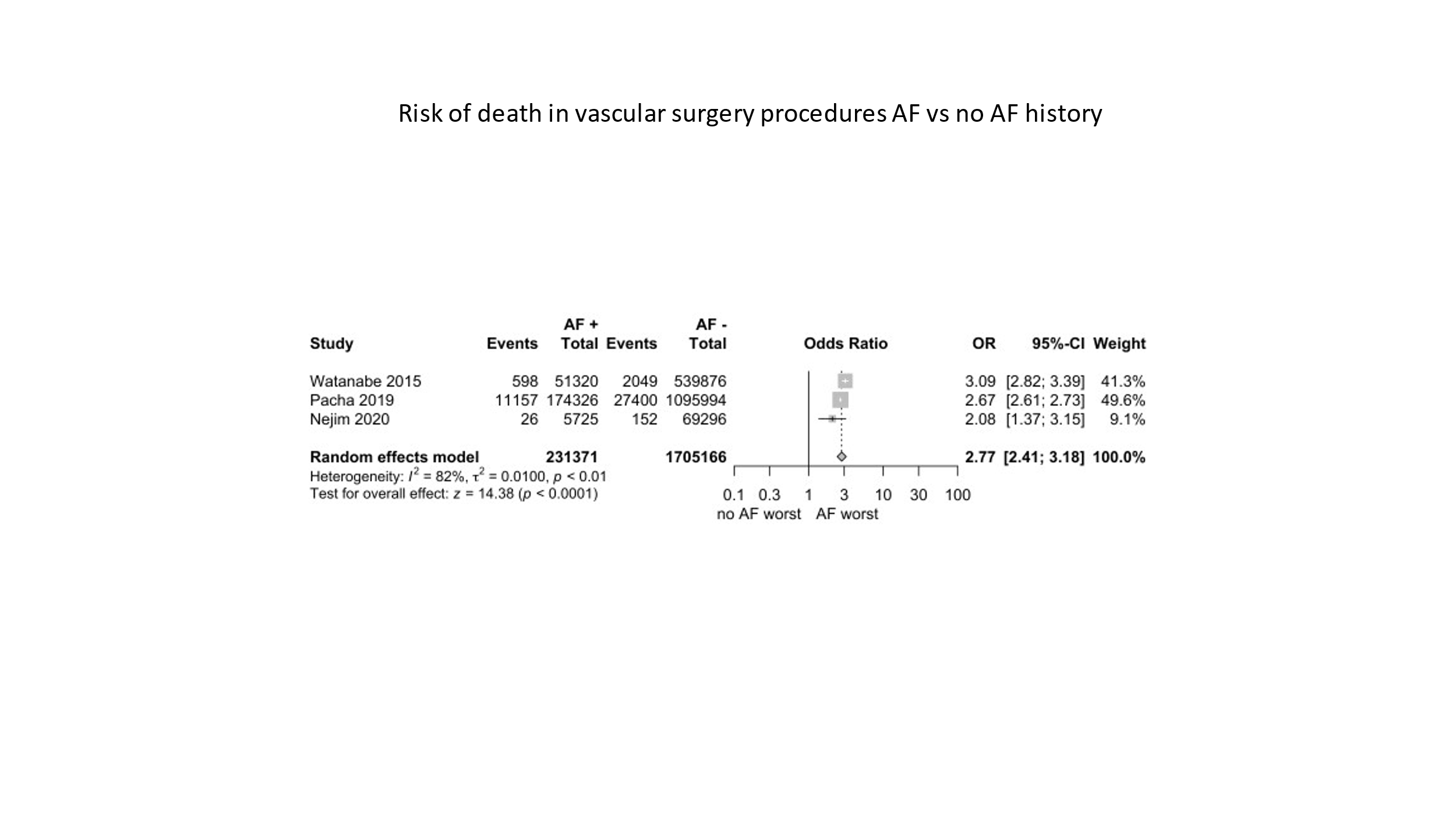


**Web Figure 6:** Risk of stroke in patients treated with endovascular surgery procedures (top panel) and in patients treated with open vascular surgery (bottom panel) according to AF presence.


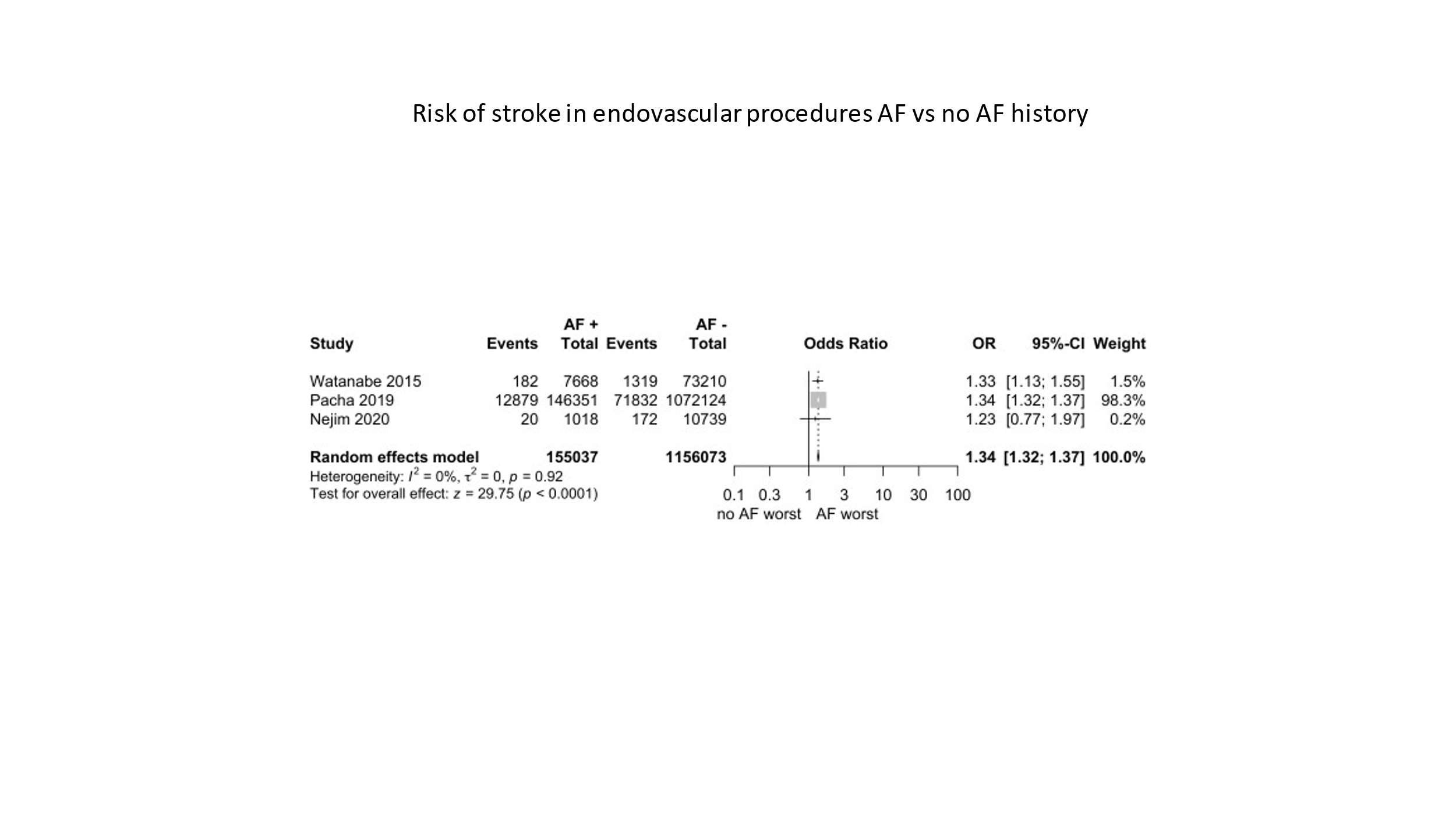


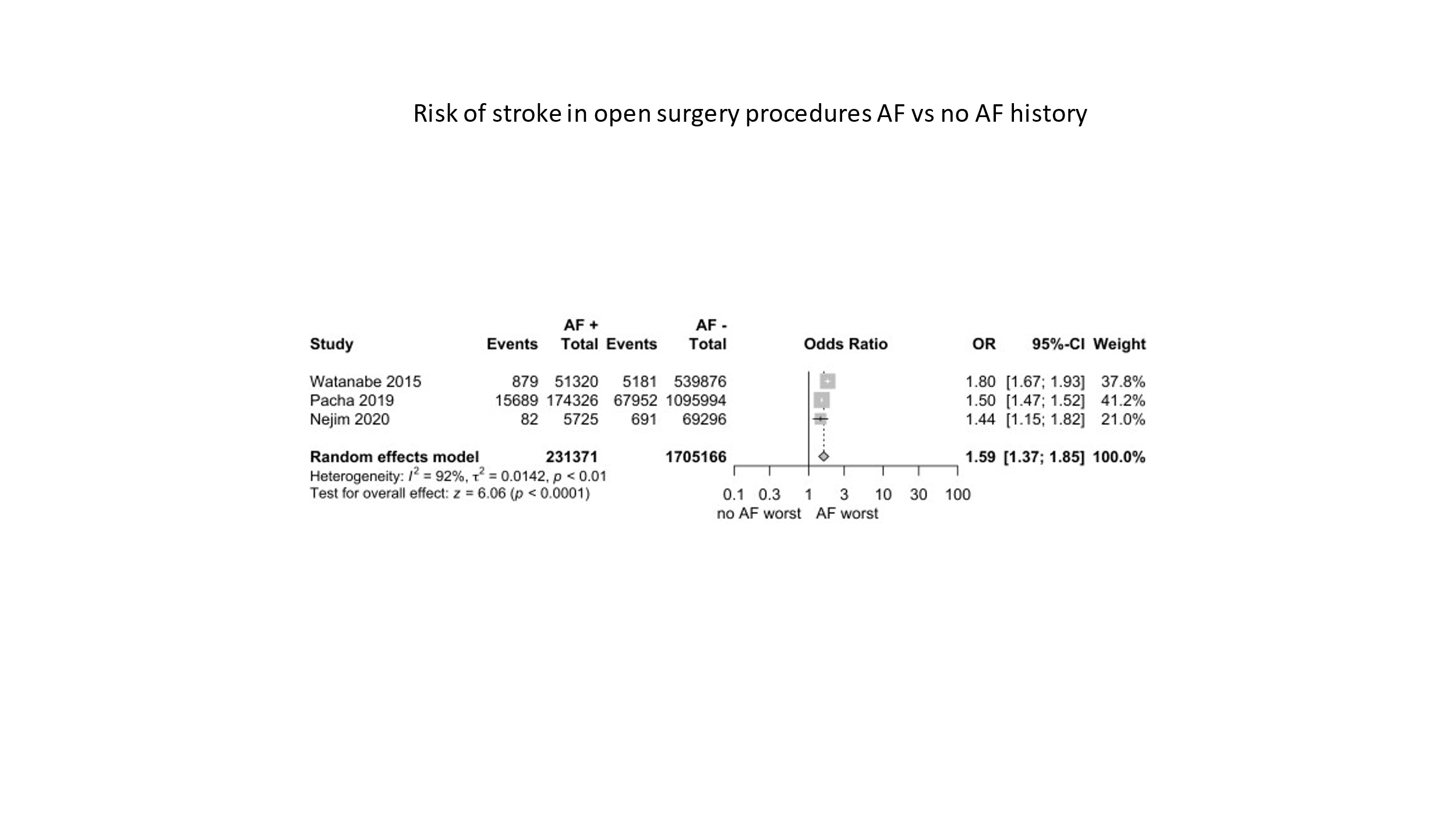


**Web Figure 7:** Leave-one-out analysis of sensitivity in studies regarding the incidence of POAF in patients treated with vascular surgery.


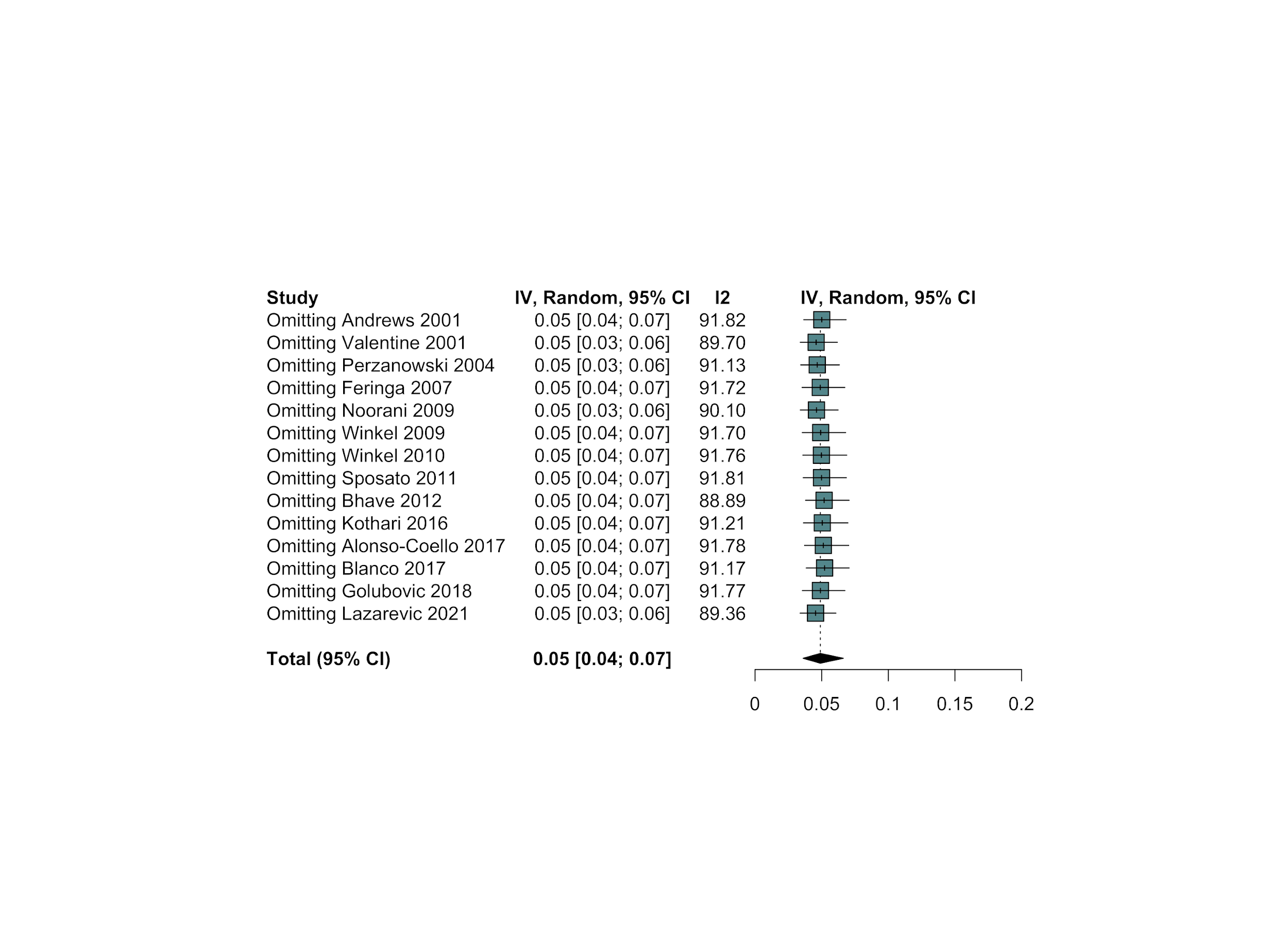


**Web Figure 8:** Funnel plot evaluating the publication bias, analyzed with Egger’s test about the incidence of POAF in vascular surgery. Of note, the statistical significance of Egger’s test.


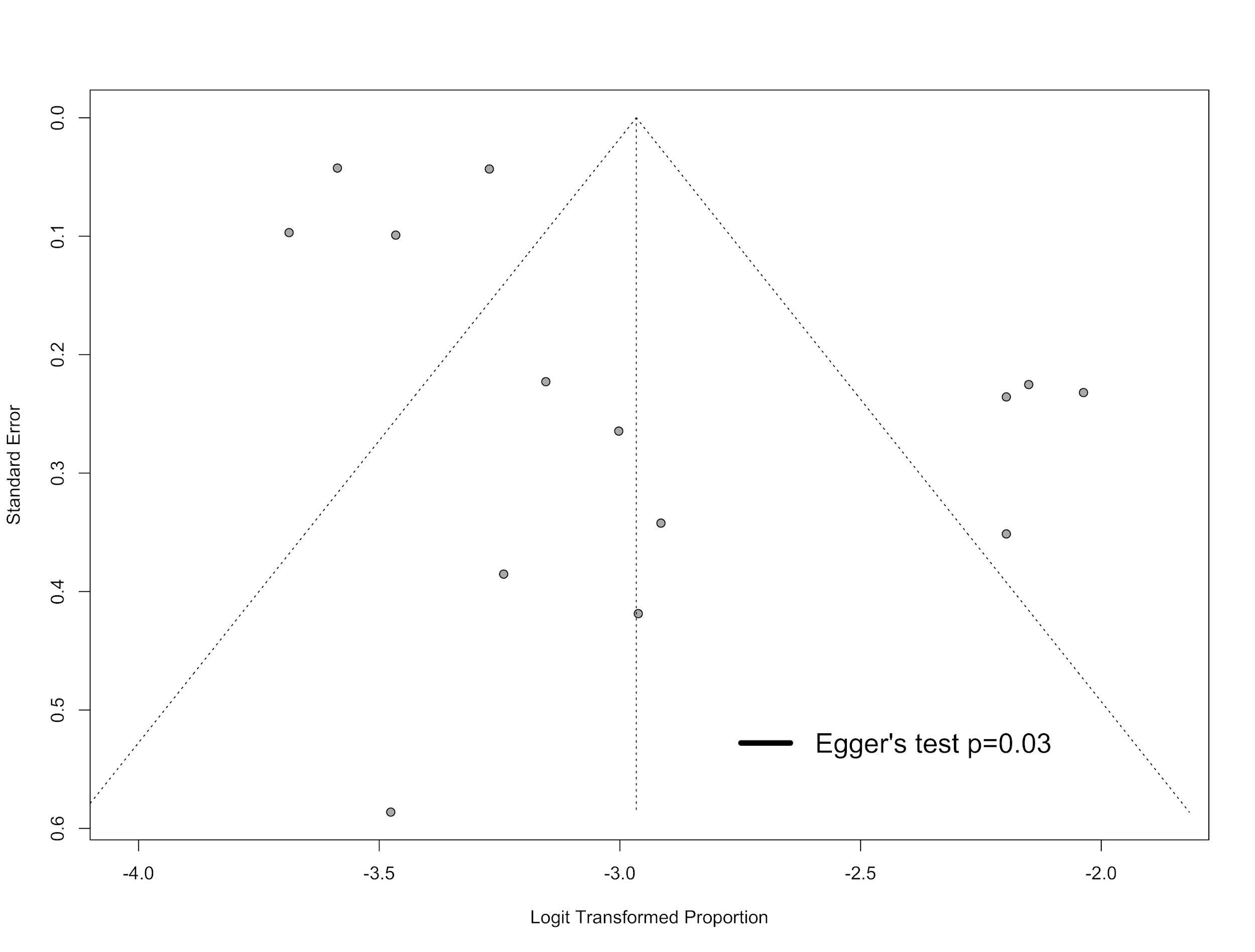


**Web Figure 9**. Pooled incidence of POAF according to the approach, i.e. open vascular surgery interventions (top) and endovascular procedures (bottom).


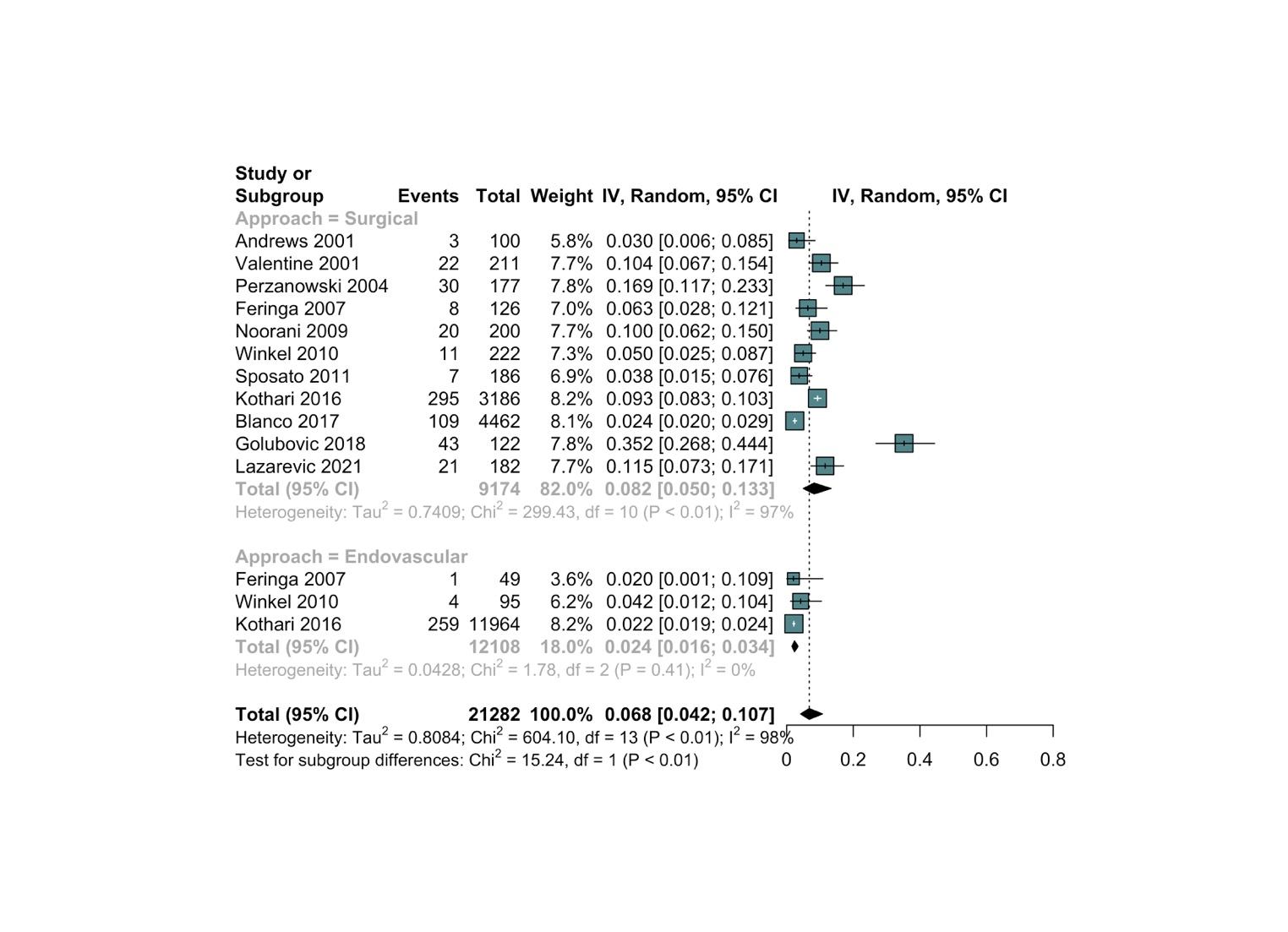


**Web Figure 10**: Pairwise metanalysis with odds related to development of POAF in endovascular procedures vs. vascular surgery interventions.


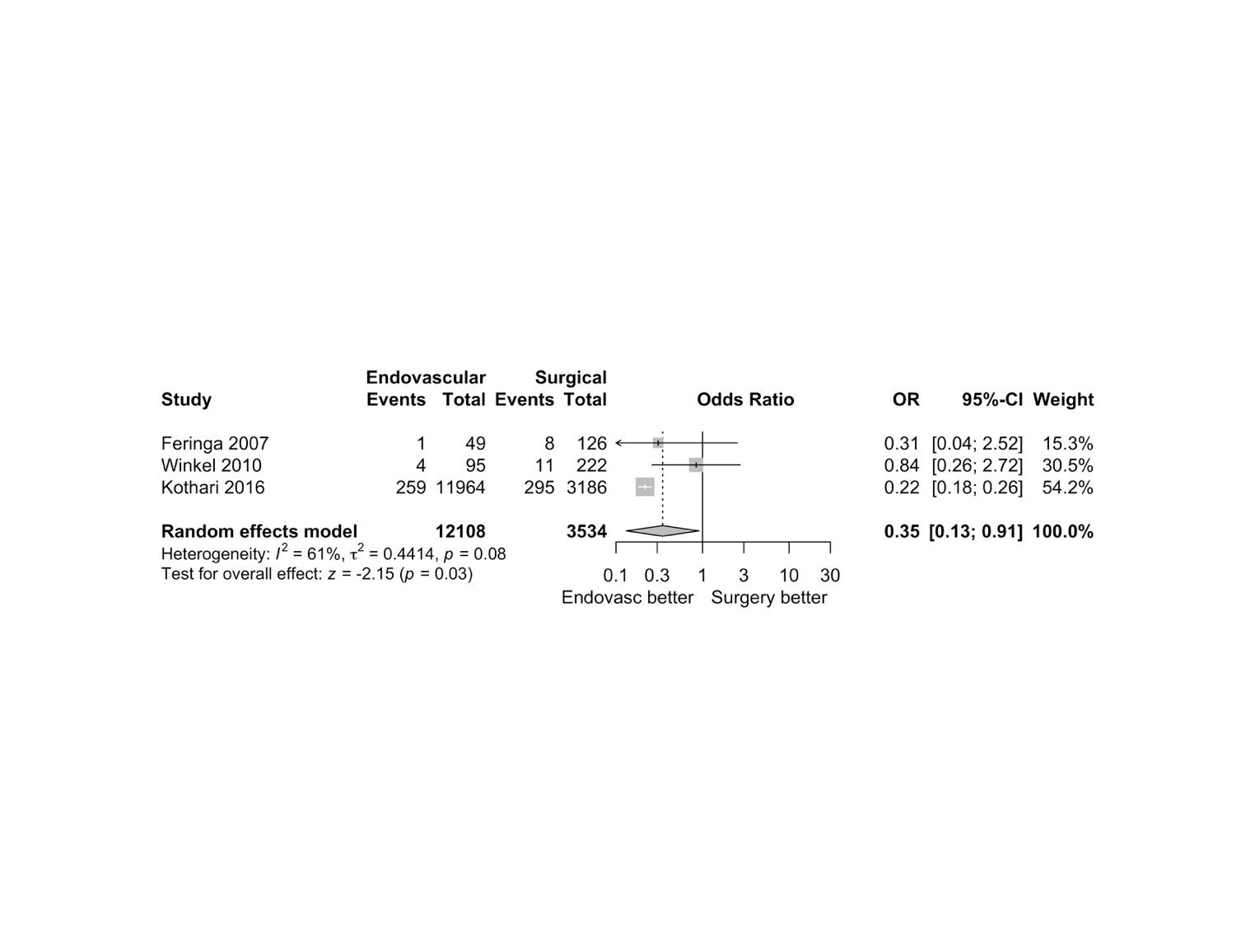


**Web Figure 11**: Metanalysis of proportion of incidence of POAF according to different sites of vascular surgery interventions


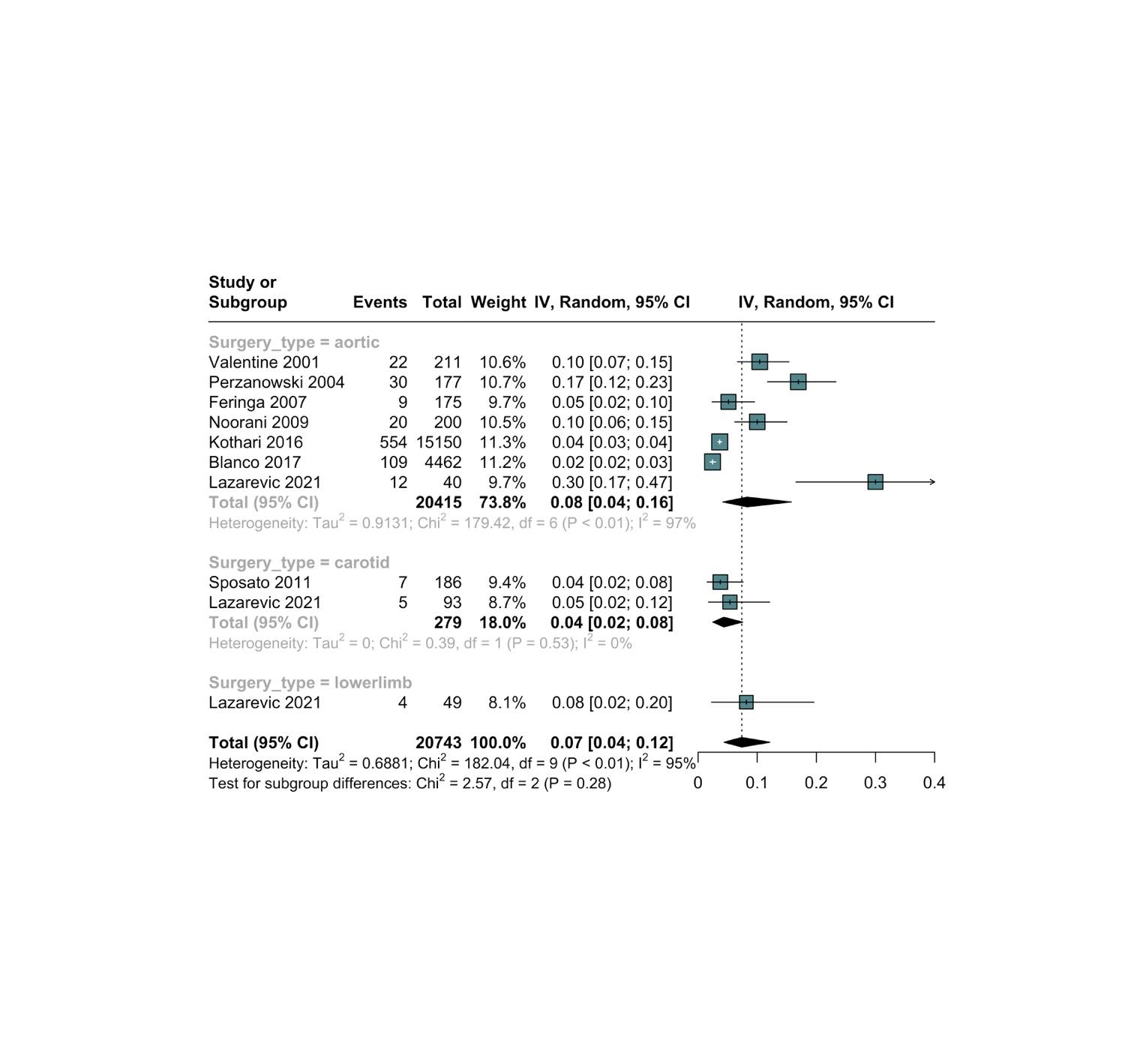

Supplement: Supplemental Digital Content [file jcarm-24-612-s001.docx]
